# A Review of the Bacterial Phosphoproteomes of Beneficial Microbes

**DOI:** 10.3390/microorganisms11040931

**Published:** 2023-04-03

**Authors:** Sooa Lim

**Affiliations:** Department of Pharmaceutical Engineering, Hoseo University, Asan-si 31499, Republic of Korea; salim0609@hoseo.edu; Tel.: +82-41-540-9591

**Keywords:** microorganisms, bacteria, proteins, post-translational modifications (PTMs), signal transduction, phosphorylation, proteomics, phosphoproteomic

## Abstract

The number and variety of protein post-translational modifications (PTMs) found and characterized in bacteria over the past ten years have increased dramatically. Compared to eukaryotic proteins, most post-translational protein changes in bacteria affect relatively few proteins because the majority of modified proteins exhibit substoichiometric modification levels, which makes structural and functional analyses challenging. In addition, the number of modified enzymes in bacterial species differs widely, and degrees of proteome modification depend on environmental conditions. Nevertheless, evidence suggests that protein PTMs play essential roles in various cellular processes, including nitrogen metabolism, protein synthesis and turnover, the cell cycle, dormancy, spore germination, sporulation, persistence, and virulence. Additional investigations on protein post-translational changes will undoubtedly close knowledge gaps in bacterial physiology and create new means of treating infectious diseases. Here, we describe the role of the post-translation phosphorylation of major bacterial proteins and review the progress of research on phosphorylated proteins depending on bacterial species.

## 1. Introduction

Bacteria play vital roles in the environment, animals, and humans and perform many essential ecological functions, such as recycling organic materials and assisting the carbon and nitrogen cycles. In contrast to plant and animal cells, bacteria are frequently subjected to continuous changes in their physical and chemical surroundings [1]. Bacterial metabolism is controlled by intracellular signals and provides the energy required for cellular activity and adaptation to different environments [2]. Bacteria rapidly adapt to various environments through post-translational modifications (PTMs) or the allosteric binding of small molecules that play a key role in metabolism. This review focuses on protein phosphorylation in PTMs. Protein phosphorylation is the most common and well-studied PTM that bacteria use to regulate protein activity and underlies bacterial protein heterogeneity. Previous studies have shown that phosphorylation is utilized more by eukaryotes than prokaryotes. Nonetheless, research efforts have resulted in the discovery of a wealth of bacterial phosphoproteins, despite the low abundances of protein modifications [3,4,5].

## 2. Bacterial Protein Phosphorylation

Bacterial protein phosphorylation subserves diverse functions in bacteria related to antibiotic resistance, such as DNA replication, metabolism, heat shock response, biofilm formation, spore formation, anti-virulence, and the production of amino acids and antibiotics. Protein homeostasis and novel protein functions can be achieved by phosphorylation, which requires components of complex cellular signal detection and conversion networks. Protein phosphorylation (His, Asp, Ser, Thr, Tyr, and Arg), glycosylation (Arg, Asn, Ser, and Thr), acetylation (Lys), acylation (Lys), lipidation (Cys), oxidation (Met), and thiolation (Cys) are the most common PTMs [6], and protein phosphorylation is one of the best understood. Amino acid residue phosphorylation can control the activity of proteins by causing structural changes in active sites and modulating protein–protein interactions. For example, in bacteria, protein phosphorylation and dephosphorylation of various amino acids provide a variety of chemical characteristics [7], stabilities, and functionalities [5]. Furthermore, protein phosphorylation plays essential regulatory roles in the cell cycle, receptor-mediated signal transduction, differentiation, proliferation, transformation, and metabolism. Two types of protein phosphorylation systems are most common in bacteria: the so-called two-component systems (TCSs), which include bacterial protein kinases, and the protein phosphorylation system, which affects serine, threonine, and tyrosine side chains.

### 2.1. Two-Component Systems (TCSs)

**Figure 1:**
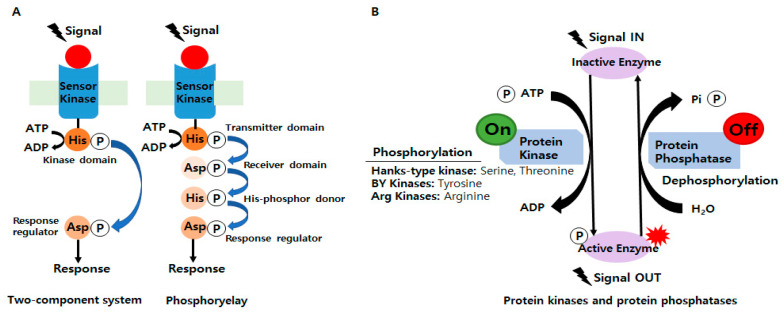
(**A**) Schematics of the prototypical two-component systems (TCSs) and phosphorelay systems in bacteria. (**B**) The overall mechanism of protein phosphorylation regulated by protein kinases and protein phosphatase.

Since the publication of a breakthrough paper on bacterial signaling in 1986, researchers have been able to share their findings on various regulatory systems. In addition, changes in protein phosphorylation and the discovery of amino acid sequence similarities in TCSs have been actively studied [8]. Bacteria sense and respond to numerous external stimuli to survive in various environments [9] and adapt to environmental changes using TCSs and phosphorelays, which are critical mediators of bacterial signal transduction (Figure 1A). In phosphorylases, a sensor kinase first transfers the phosphoryl group to a response regulator with a conserved aspartate domain but no output domain, which is a more complicated type of TCS [10]. TCSs comprise at least two proteins: a sensor kinase and a response regulator. It was predicted that bacteria exhibit signaling phosphorylation mainly at His and Asp residues [11]. The former senses external stimuli, while the latter alters the expression profiles of bacterial genes required for survival and adaptation [12]. In other words, TCSs play a significant role in the general regulatory network by integrating external signals and information from stress pathways, central metabolism, and global regulators [13].

For example, the PhoQ/PhoP TCS detects several host stimuli, such as extracellular magnesium restriction, low pH, cationic antimicrobial peptides, and osmotic stress [5,14]. TCSs are critical for the coordinated expression of virulence factors and, in some situations, for bacterial viability and proliferation. Several studies have shown that TCSs regulate virulence and antibiotic resistance in pathogenic bacteria [9,15,16,17,18,19,20,21,22,23,24,25,26]. Furthermore, the mechanisms of specific TCSs inhibitors differ from those of existing antibiotics and might facilitate the development of effective drugs against drug-resistant bacteria [5,12,15,27,28]. Serine/threonine kinases, which usually have multiple substrates, can also phosphorylate TCS response regulators [5]. The reported regulatory modes of five TCSs in Escherichia coli (*E. coli*) exhibited novel relationships: MG1655, BaeSR, and CpxAR are stimulated by ethanol stress; KdpDE and PhoRB are induced by low levels of potassium and phosphate, respectively; and ZraSR is stimulated by zinc [29]. Human TCS genes have been compared to TCS genes in *Francisella tularensis*, a Gram-negative bacterium that causes disease in various hosts [13]. Furthermore, a recent report showed that TCSs utilize multiple mechanisms, such as cross-regulation, to integrate and coordinate input stimuli to control biofilm formation [30,31,32,33,34,35,36].

### 2.2. Involvements of Ser/Thr/Tyr/Arg Kinases in Bacterial Signaling and Regulation

Unlike TCS histidine kinases, which usually phosphorylate one response regulator, Hanks-type kinases and BY kinases tend to phosphorylate multiple protein substrates (Figure 1B) [5]. In phosphorylases, a sensor kinase first transfers the phosphoryl group to a response regulator with a conserved aspartate domain but no output domain, which is a more complicated type of TCS [10]. Phosphoproteomic surveys over the past decade in phylogenetically diverse bacteria have identified numerous proteins phosphorylated at Ser/Thr (ST) residues [11]. Furthermore, Tyr phosphorylation regulates several cellular processes in bacteria [37,38]. Historically, the phosphorylation of ST residues in bacterial proteins was first identified by pioneering experiments in the 1970s. However, by the early 1980s, most research focused on TCSs [39], HPr kinase/phosphorylases [40,41,42], and the isocitrate dehydrogenase (Icd) kinase/phosphatase system [43,44,45,46]. As a result, researchers arrived at the premature conclusion that eukaryotes possess only Ser/Thr/Tyr (STY) kinases and that bacteria possess mainly His/Asp kinases. On the other hand, with the advent of genomic sequencing in the 1990s, genes encoding ST kinases were widely identified in bacterial genomes [47], and this apparent complexity presented the challenge of identifying the substrates of these bacterial kinases. Comprehensive searches for proteins containing phosphorylated STY residues in *E. coli*, *Bacillus subtilis* (*B. subtilis*), and *Lactococcus lactis* (*L. lactis*) in 2007 gave rise to bacterial phosphoproteomics [3,4,47,48]. Since then, hundreds of homologous TCSs have now been identified in eukaryotic organisms. Reversible phosphorylation of STY residues has also been found in many prokaryotes identified as having equal or greater numbers of STYs than eukaryotes [47,49,50,51]. For example, numerous eukaryotic ST kinases that participate in complex signaling pathways help regulate the *Myxococcus xanthus* (*M. xanthus*) life cycle [52]. In addition, bacterial kinases with catalytic domains may share structural and functional homology with eukaryotic ST kinases [53,54]. Knowledge of protein kinases/phosphatases has expanded as researchers have further defined bacterial evolutionary conservation. Therefore, the roles of bacterial proteins containing phosphorylated STY residues corresponding to protein kinases/phosphatases during signal transduction need to be fully understood. Bacterial protein phosphorylation, which performs a variety of functions including antibiotic resistance, DNA replication and metabolism, heat shock response, biofilm formation, sporulation, and antitoxicity, is continuously investigated [50,55,56,57,58,59,60]. Several years ago, new evidence suggested that arginine phosphorylation and dephosphorylation are key regulators in bacteria, which implied these modifications might also be important physiologically [61,62,63,64,65,66,67,68,69,70,71].

## 3. Bacterial Phosphoproteomics

Phosphorylation represents a dynamic change, and phosphoproteins are commonly present at very low levels. As a result, precise and sensitive techniques are needed for phosphoproteome analysis. A large body of phosphoproteomic research has been conducted using mass spectrometry techniques in conjunction with specific phosphor-enrichment techniques [1]. Additionally, specific tools have been developed to study the many substrates of STY kinases. Traditional phosphoproteomics, utilized in bacteriology before 2007, relied on 1D- and 2D-gel 32P-radiolabeling or Western blotting with immunodetection followed by low-resolution mass spectrometry. Although 2D gel electrophoresis enables the simultaneous separation of hundreds of proteins, this tool has poor reproducibility, under-represents low-abundance and hydrophobic proteins, and has a poor dynamic range [72]. Furthermore, the ability of 2D gel electrophoresis to resolve integral membrane proteins is limited because of protein aggregation during the first isoelectric-focusing (IEF) migration, and this technique is particularly ineffective at identifying sites of phosphorylation. However, the efficient enrichment of phosphorylated peptides before mass spectrometry has revolutionized phosphoproteomics, and since 2007, high-resolution mass spectrometry coupled with gel-free analysis has led to the elucidation of site-specific STY bacterial phosphoproteomes in many bacterial species.

### 3.1. Phosphoproteome Analysis of Beneficial Microorganisms

The first phosphoproteome studies suggested [3,4,48] that phosphorylations are critical regulatory events of bacterial metabolism and showed that bacterial phosphoproteins and phosphorylated residues are associated with evolutionary conservation. Hundreds of biological meaningful phosphorylation sites in bacteria had been found by 2019 [73,74,75,76,77,78,79,80,81,82,83,84,85,86,87,88]. The immobilized metal ion affinity chromatography (IMAC) phosphopeptide enrichment technique was used to identify more than 2000 phosphorylated proteins [81,82,89]. In 2021, 19 phosphoproteomic studies on bacteria were reported, and the phosphoproteomes of 14 bacteria were analyzed and biologically interpreted [54,71,90,91,92,93,94,95,96,97,98]. Increasing evidence shows that bacterial phosphorylation sites are as versatile as those of eukaryotes. Furthermore, many studies have emphasized the utilities of protein phosphorylation events and their associated kinases/phosphatases for elucidating the associated physiological processes. Table 1 lists phosphoproteomics studies conducted on 35 bacterial species since the start of phosphoprotein research in 2007. In addition to STY phosphorylation in bacteria, recent research efforts have also studied the phosphorylation of histidine (His, the most abundant bacterial protein) [71,81,82,89,93,99,100] and arginine, which plays a significant role in bacteria [71,82,101]. Prior to 2014, experiments on sub-stoichiometric phosphopeptide enrichment were performed under strong acidic conditions, which explains why phosphorylated histidine residues were difficult to detect. However, phosphorylated His proteins can now be identified using recently developed methods [81,89]. Furthermore, several new methods have been devised to analyze arginine since it was discovered that arginine phosphorylation plays an important role in Gram-positive bacteria [70,71]. The information provided in Table 1 may be expansive, but it provides comprehensive reference information on research techniques and trends for those studying phosphorylated proteins. Furthermore, it provides information for researchers studying specific bacteria regarding the detection of phosphorylated proteins.

### 3.2. Phylogenetic Diagram of Beneficial Microorganisms

**Figure 2:**
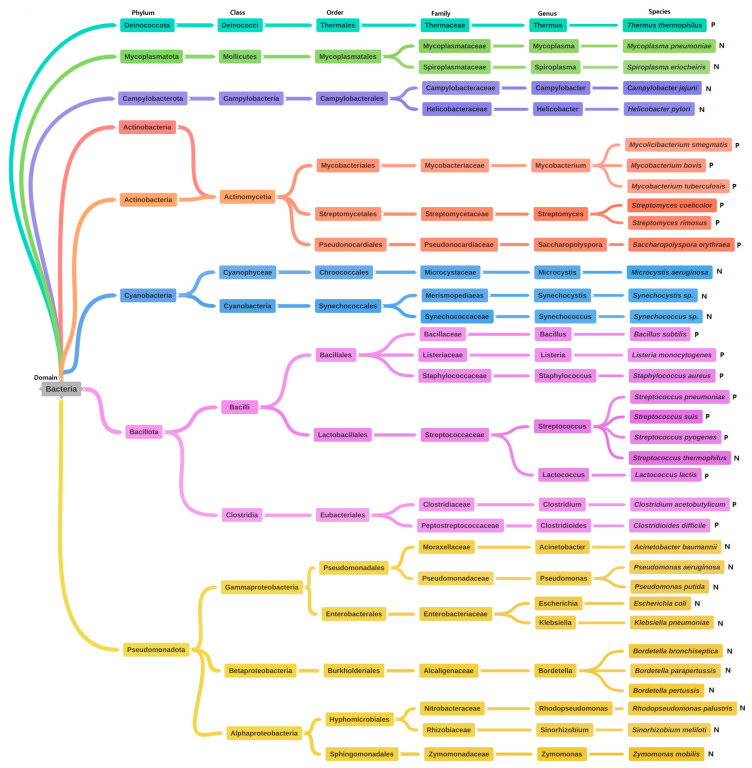
Taxonomic classifications of beneficial microorganisms subjected to phosphorylation studies. (P): Gram-positive bacteria and (N): Gram-negative bacteria.

This review also provides an overview of useful microorganisms subjected to phosphoproteomic studies. Figure 2 lists the 35 bacterial species investigated, divides them into 8 phyla, 11 classes, 16 orders, 24 families, and 26 genera, and classifies them as Gram-positive bacteria (P, n = 16) or Gram-negative bacteria (N, n = 19).

Mycobacterium is a genus in the phylum Actinomycetota and is assigned its own family, Mycobacteriaceae. This genus includes pathogens known to cause serious diseases in mammals and tuberculosis in humans. Biochemical and signaling pathways involved in pathogenicity were investigated in virulent H37Rv and non-virulent H37Ra [122] strains to investigate protein phosphorylation networks using clinical isolates of *M. tuberculosis* [79]. In addition, a phosphoprotein study was undertaken to understand how antibiotic resistance develops [80] and to obtain insights into the regulatory roles of phosphoproteins in Mycobacterium growth and development [78,84].

Antibiotics, such as actinorhodin, methylenomycin, undecylprodigiosin, and perimycin, are produced by different Streptomyces strains [108]. Immobilized zirconium (IV) affinity chromatography and mass spectrometry were used to discover more phosphoproteins [95] and understand the roles of phosphoproteins in *Streptomyces coelicolor (S. coelicolor)* [106]. Bacterial differentiation and secondary metabolic activation in *S. coelicolor* were recently investigated using a quantitative mass spectrometry-based/proteomics/ phosphoproteomics approach [85].

Bacilli is a class of Gram-positive aerobic bacteria that includes the orders Bacillaes and Lactobacillales. Bacillales are a representative genus that includes Bacillus, Listeria, and Staphylococcus, and *Bacillus subtilis* (*B. subtilis*) is used as a model for research on bacterial cell differentiation and chromosome replication. This bacterium is used commercially to synthesize large amounts of enzymes [125,126,127], and *B. subtilis* 168 has been reported to contain a number of biologically significant phosphoproteins [3,54,77,93,115]. *Listeria monocytogenes* (*L. monocytogenes*) is a pathogenic soil bacterium, and after 143 phosphorylation sites [107] were discovered in this bacterium, an automated STY phosphopeptide enrichment method was devised to investigate the relationship between protein phosphorylation, toxicity mechanisms, and carbon metabolism, and as a result, 420 phosphorylation sites were detected [54]. Phosphorylated proteins in *Staphylococcus aureus* have been found to be associated with pathogenicity and virulence. An effective phosphopeptide enrichment technique was developed to understand how protein phosphorylation affects complex signaling networks associated with pathogenicity, and eight proteins phosphorylated on arginine residues have been identified [93,101]. Research has shown that arginine phosphorylation plays a significant and relevant role in metabolism [71]. Streptococcus is a genus of Gram-positive coccus or spherical bacteria belonging to the family Streptococcaceae, within the order Lactobacillales in the phylum Bacillota [128]. The pathogenic bacterium *Streptococcus pneumoniae*, which plays an essential regulatory role in complex protein phosphorylation metabolic pathways and bacterial virulence, has been studied [104]. A systematic study of ST kinases and phosphatases of the pathogen *Streptococcus suis* (*S. suis*) was performed using comparative phenotypic, proteomic, and phosphoproteomic assays [91]. In addition, studies were conducted to identify the proteins and pathways tagged by STY phosphorylation in *Streptococcus thermophilous* (*S. thermophilous*), a lactic acid bacterium used extensively for dairy fermentation [56]. The class Clostridia includes *Clostridium acetobutylicum* (*C. acetobutylicum*), which produces butanol, and *Clostridioides difficile* (*C. difficile*), a well-known enteropathogen. The extent and nature of phosphorylation in the Gram-positive enteropathogen *C. difficile* have not been well characterized. PTMs have been studied [98], and a promising study was conducted to provide detailed mapping of kinase–substrate relationships in *C. difficile* to identify novel biomarkers and therapeutic targets [96].

Cyanobacteria of the species *Microcystis aeruginosa* (*M. aeruginosa*) can play a crucial role in synthesizing cyanotoxins, particularly the potent liver poisons known as microcystins, and thus, the relation between toxin generation and phosphoproteomic profiles was studied in M. aeruginosa [123]. Cyanobacteria, such as *Synechocystis* sp., play important ecological roles. Ser, Thr, and Tyr phosphorylation contribute to the basic mechanisms that regulate homeostasis in cyanobacteria [113,121].

Thermus is a genus of thermophilic bacteria belonging to the Deinococcota phylum, and the research, biotechnological, and industrial potentials of thermostable enzymes isolated from members of the Thermus genus are of great interest. The phosphoproteins of *Thermus thermophilus* (*T. thermophilus*) HB8 identified using phosphoproteome analysis are involved in various cellular processes [112]. In a phosphoproteomic study on *T. thermophilus* HB27, phosphorylation affected PilF phosphorylation on type IV pilus and biofilm formation [75].

*Mycoplasma pneumoniae* (*M. pneumoniae*) belongs to the Mollicutes class and is a diminutive bacterium capable of host-independent life. In humans, *M. pneumoniae* causes mycoplasma pneumonia, a form of atypical bacterial pneumonia related to cold agglutinin disease. This bacterium exhibits little regulation of gene expression, which is why its phosphorylated proteins are biologically important [74].

Gammaproteobacteria, Alphaproteobacteria, and Betaproteobacteria are classes of bacteria in the phylum Pseudomonadota. Pseudomonas, Moraxella, and Acinetobacter species are pathogens that can cause disease in humans, animals, and plants. *Acinetobacter baumannii* (*A. baumannii*) can be pathogenic in individuals with a weakened immune system, and is garnering attention as a cause of nosocomial infections [129]. In one study, the STY phosphoprotein properties of two *A. baumannii* reference strains (ATCC17978) and a highly invasive, multidrug-resistant clinical isolate (Abh12O-A2) were compared, and the results obtained highlighted the roles of phosphoproteins in pathogenicity and drug resistance [76]. The roles of AmpC β-lactamase phosphorylation were also compared in a mipenem-susceptible Acinetobacter baumannii SK17-S and resistant SK17-R strain [100]. *E. coli* is a rod-shaped, Gram-negative, facultative anaerobic organism that can be grown and cultured easily and inexpensively in a laboratory environment [130], and studies have confirmed that specific phosphorylated bacterial proteins are involved in translational arrest, growth inhibition, and the induction of physiological dormancy [83]. Phosphoproteomics studies have generated large datasets of bacterial phosphorylated protein with the aim of understanding cellular processes [4,77,83,114,130]. Approximately 30% of *Klebsiella pneumoniae* (*K. pneumoniae*) strains naturally present in soil can fix nitrogen in anaerobic environments, and *K. pneumoniae* has been shown to increase crop yields via nitrogen fixation [131]. Encapsulated *K. pneumoniae*, an important pathogen in nosocomial infections, contains protein-tyrosine kinases and phosphatases, which are viewed as keys to deciphering its virulence [102]. An enrichment process was developed to identify more phosphopeptides in a single bacterial sample [77]. *Rhodopseudomonas palustris* (*R. palustris*) has a variable metabolism and can grow in photoheterotrophic and chemoheterotrophic conditions. This species is used to control carbon metabolism by phosphorylation at the threonine residue and produce hydrogen, lipids, and thus butanol [111]. In addition, the phosphoproteome of *Bordetella pertussis*, *bronchiseptica*, and *parapertussis* were characterized, and their potential roles in Bordetella biology and virulence were examined. Bordetella are pathogens that cause whooping cough or diseases resembling whooping cough. Globally, bordetella infections have increased, necessitating a greater understanding of these diseases and the developments of novel medications and vaccines [92].

## 4. Conclusions

Bacteria play vital roles in the environment, animals, and humans. Bacterial protein phosphorylation serves diverse functions in bacteria, such as antibiotic resistance, DNA replication and metabolism, heat shock response, biofilm formation, spore formation, anti-virulence, and the production of amino acids and antibiotics. Bacteria contain extremely small amounts of phosphoproteins, but despite this, phosphoproteins influence essential cellular processes. Research on two-component systems (TCSs) and the protein phosphorylated at Ser/Thr/Tyr (STY) residues began in 2008, and hundreds of biologically relevant phosphorylation sites have since been discovered in bacteria. Furthermore, increasing evidence indicates that bacterial phosphorylation sites are as versatile and rich as those in eukaryotes. Advances in proteomic technology have resulted in the discovery of many bacterial phosphoproteins, and advances in LC-MS/MS technology and phosphopeptide enrichment over the last 20 years have enabled the study of large datasets of Ser/Thr/Tyr/Arg phosphopeptides in bacteria. Prior to 2014, experiments on sub-stoichiometric phosphopeptide enrichment were done under strong acidic conditions, which explains why phosphorylated histidine residues were difficult to detect. However, phosphorylated His proteins can now be identified using recently developed methods [81,89]. Furthermore, several new methods have been devised to analyze arginine since it was discovered that arginine phosphorylation plays an important role in Gram-positive bacteria [70,71]. Because technological advances have enabled researchers to determine the biological significances of individual microbes, we undertook this review to summarize studies on the phosphorylation of proteins and the phylogeny of microbes. Table 1 provides a summary of the status of Ser/Thr/Tyr/His/Arg phosphorylated protein analyses conducted on beneficial microorganisms, and Figure 2 summarizes why researchers studied these microorganisms and findings of biological significance. Although this information may be somewhat expansive, it provides comprehensive reference information on research techniques and trends for those studying phosphorylated proteins. Furthermore, it provides information for researchers studying specific bacteria regarding the detection of phosphorylated proteins. This review article was also produced in part to help researchers find information on the biological significance of phosphoproteins and provide information on research ideas and trends.

## Figures and Tables

**Table 1:** Bacterial Ser/Thr/Tyr/His/Arg phosphoprotemics studies.

Organism	Strain	Year	P-pro. (ea)	P-pep. (ea)	P-site (ea)	Ser (%)	Thr (%)	Tyr (%)	Arg (%)	His (%)	Refs.
*C. jejuni*	11168	2007	36	58	35	30.3	72.7	9.1			[73]
*B. subtilis*	168	2007	78	103	78	69.2	20.5	10.3			[3]
*L. lactis*	Il1403	2008	63	102	79	46.5	50.6	2.7			[48]
*E. coli* K12	MG1665	2008	79	105	81	67.9	23.5	8.6			[4]
*K. pneumoniae*	K2044	2009	81	117	93	31.2	15.4	25.8			[102]
*P. putida*	MK25	2009	40	56	53	52.8	39.6	7.5			[103]
*P. aeruginosa*	PAO1	2009	23	57	55	52.7	32.7	14.5			[103]
*M. pneumoniae*	M129	2010	63	16	16	53.3	46.7	0			[74]
*S. pneumoniae*	D39	2010	84	102	163	47.2	43.8	9			[104]
*M. tuberculosis*	H37Rv	2010	301	381	506	40	60	0			[105]
*S. coelicolor*	A3(2)	2010	40	44	46	34.1	52.3	13.6			[106]
*L. monocytogenes*	EGDe	2011	112	155	143	93	43	7			[107]
*S. coelicolor*	M145	2011	127	260	289	46.8	48	5.2			[108]
*H. pylori*	26695	2011	67	80	124	42.8	38.7	18.5			[109]
*C. acetobutylicum*	ATCC824	2012	61	82	107	42	47.6	10.6			[110]
*R. palustris* ^(Ch)^	CGA010	2012	54	100	63	63.3	16.1	19.4			[111]
*R. palustris* ^(Ph)^	CGA010	2012	42	74	59	58.9	23.2	17.9			[111]
*T. thermophilus*	HB8	2012	48	52	46	30	12	4			[112]
*T. thermophilus*	HB27	2013	53	93	67	57	36	7			[75]
*Synechococcus* sp.	PCC7002	2013	245	280	410	43.9	42.4	13.6			[113]
*E. coli* K12	BW25113	2013	133	150	108	75.9	16.7	7.4			[114]
*S. aureus*	COL	2014	108		68	50	25	15	10		[101]
*A. baumannii*	AbH120A2	2014	70		80	70.8	25.2	3.8			[76]
*A. baumannii*	17978	2014	41		48	68.9	24.1	5.2			[76]
*B. subtilis*	168	2014		177	155	74.6	18.6	7.3			[115]
*S. erythraea*	NRRL2338	2014	88	109		47	45	8		5.3	[99]
*P. aeruginosa*	PA14	2014	28	43	59	49	24	27			[116]
*L. monocytogenes*	∆PrfA	2014	191	256	242	155	75	12			[117]
*S. meliloti*	CCBAU	2015	77	88	96	63	28	5			[118]
*B. subtilis*	Spore	2015	124		155	77.41		22.6			[119]
*B. subtilis*	168	2015	175	441	339	74.8	17.7	7.1			[77]
*E. coli* K12	BW25113	2015	392	1212	1088	69.5	21.8	7.7			[77]
*E. coli* K12	MG1655	2015	71	82							[120]
*K. pneumoniae*	K2044	2015	286	663	559	72.9	13.7	12.9			[77]
*Synechocystis* sp.	PCC 6803	2015	188	242	262						[121]
*M. tuberculosis*	SAW5527	2015	214	303	414	38	59	3			[79]
*M. smegmatis*	mc2155	2015	2462	464	185	39.5	57.1	3.5			[78]
*M. bovis BCG*	1173P2	2015	1765	402	442	35	61.6	3.1			[78]
*M. tuberculosis*	B0/W148	2016	132	180	191	22	76	2			[80]
*A. baumannii*	SK17-S	2016	248	351	410	47	27.6	12.4		4.9	[100]
*A. baumannii*	SK17-R	2016	211	240	285	41.4	29.5	17.5		4.9	[100]
*M. tuberculosis*	H37Ra	2017	257		512	29	68	3			[122]
*M. smegmatis*	mc2155	2018	154	222	242	24.8	74.0	1.2			[84]
*M. aeruginosa*	FACHB-469	2018	37		59						[123]
*M. aeruginosa*	FACHB-905	2018	18		26						[123]
*S. coelicolor*	M145	2018	48	92	85	50.6	47.4	2			[85]
*E. coli* K12	MG1665	2018	632	1178	1183						[83]
*E. coli* K12	W3110	2018	861		2446	57.2	25.3	8.5		9	[81]
*E. coli* K12	W3110	2018	781	2057	2129	1220	501	162		246	[89]
*E. coli* K12	W3110	2018			2248	56	20	13	5	5	[82]
*Z. mobilis*	ZM4,31821	2019	125		177	73	21	6			[124]
*S. thermophilus*	LMD9	2019	106	410	161	43	33	23			[56]
*S. eriocheiris*	M207170	2019	245		465						[86]
*E. coli* K12	1655, ∆yea	2021	83	127		67.7	28.3	3.9			[94]
*B. subtilis*	168	2021	146	283	267	73	12.7	7.5		6.7	[93]
*S. aureus*	USA300	2021	859	3800	3771	55.2	29.6	7.3		7.8	[93]
*B. subtilis*	168	2021	153		214	67	28	5			[54]
*S. pyogenes*	M1	2021	205		449	41	55	4			[54]
*L. monocytogenes*	EGDe	2021	241		420	56	35	9			[54]
*B. pertussis*	L1423	2021	45	53	54	72	17	11			[92]
*B. bronchiseptica*	RB50	2021	23	28	29	69	21	10			[92]
*B. parapertussis*	12822	2021	42	50	50	80	12	8			[92]
*M. bovis*	BCG, ΔPknG	2021	914	1371	1401	85.3	13.4	1.3			[90]
*S. suis*	WT, Δstp	2021	50		73						[91]
*S. suis*	WT, Δstk	2021	67		87						[91]
*S. aureus*	NE98, ΔSdrE	2022	953		4407	45.5	24	5	20.2	5.4	[71]
*S. aureus*	NE217, ΔStk1	2022	903		3779	48.1	22	6.7	18	5.2	[71]
*S. aureus*	NE1919, ΔStp1	2022	951		4085	40.2	21.2	6.1	26	6.5	[71]
*C. difficile*	630WT	2022	700	2994	1759	75	20	5			[98]
*C. difficile*	630WT, Δ erm	2022	504	1061	117	76.6	17.8	5.6			[96]
*S. rimosus*	G7, 10970	2022	230	273	417	41.3	53.5	5.3			[97]
*S. coelicolor*	A3(2)	2022	187	351	361	41	56.2	2.8			[95]

Experimental phosphoproteome coverage is shown in terms of identified phosphorylated proteins (P-pro.), phosphopeptides (P-pep.), and phosphorylated sites (P-site). Data were extracted from research publications or databases. Blank areas: not reported; (Ch) chemoheterotrophic growth; (Ph) photoheterotrophic growth.

## Data Availability

Not applicable.
